# Blood‐based detection of *RAS* mutations to guide anti‐EGFR therapy in colorectal cancer patients: concordance of results from circulating tumor DNA and tissue‐based *RAS* testing

**DOI:** 10.1002/1878-0261.12023

**Published:** 2017-01-20

**Authors:** Wolff Schmiegel, Rodney J. Scott, Susan Dooley, Wendy Lewis, Cliff J. Meldrum, Peter Pockney, Brian Draganic, Steve Smith, Chelsee Hewitt, Hazel Philimore, Amanda Lucas, Elva Shi, Kateh Namdarian, Timmy Chan, Danilo Acosta, Su Ping‐Chang, Andrea Tannapfel, Anke Reinacher‐Schick, Waldemar Uhl, Christian Teschendorf, Heiner Wolters, Josef Stern, Richard Viebahn, Helmut Friess, Klaus‐Peter Janssen, Ulrich Nitsche, Julia Slotta‐Huspenina, Michael Pohl, Deepak Vangala, Alexander Baraniskin, Barbara Dockhorn‐Dworniczak, Susanne Hegewisch‐Becker, Philippe Ronga, Daniel L. Edelstein, Frederick S. Jones, Stephan Hahn, Stephen B. Fox

**Affiliations:** ^1^ Department of Internal Medicine Medical University of Bochum Hospital Germany; ^2^ Pathology North John Hunter Hospital New Lambton Heights Australia; ^3^ Hunter Medical Research Institute Newcastle Australia; ^4^ The Faculty of Medicine and Health University of Newcastle Australia; ^5^ Division of Surgery John Hunter Hospital New Lambton Heights Australia; ^6^ Peter MacCallum Cancer Centre and the University of Melbourne Melbourne Australia; ^7^ Institute of Pathology University of Bochum Hospital Germany; ^8^ Division of Hematolgy and Oncology Department of Internal Medicine St. Josef Hospital Ruhr‐University Bochum Germany; ^9^ Department of Visceral and General Surgery St. Josef Hospital Ruhr‐University Bochum Germany; ^10^ Department of Internal Medicine St. Josefs‐Hospital Dortmund Germany; ^11^ Department of Visceral and General Surgery St. Josefs‐Hospital Dortmund Germany; ^12^ Department of Visceral and General Surgery Medical University of Bochum Hospital Germany; ^13^ Department of Surgery University Hospital Klinikum rechts der Isar Technical University Munich Germany; ^14^ Department of Pathology Technical University Munich Germany; ^15^ Department of Molecular GI Oncology University of Bochum Hospital Germany; ^16^ Centre of Pathology Kempten Germany; ^17^ MVZ Humangenetics and Laboratory Medicine Martinsried Germany; ^18^ Centre of Haemato‐Oncology Hamburg Germany; ^19^ Global Medical Affairs Oncology Merck KGAa Darmstadt Germany; ^20^ Medical Scientific Affairs Sysmex Inostics Inc. Mundelein IL USA

**Keywords:** anti‐EGFR therapy, CRC, ctDNA, plasma, RAS mutations

## Abstract

An accurate blood‐based *RAS* mutation assay to determine eligibility of metastatic colorectal cancer (mCRC) patients for anti‐EGFR therapy would benefit clinical practice by better informing decisions to administer treatment independent of tissue availability. The objective of this study was to determine the level of concordance between plasma and tissue *RAS* mutation status in patients with mCRC to gauge whether blood‐based *RAS* mutation testing is a viable alternative to standard‐of‐care *RAS* tumor testing. *RAS* testing was performed on plasma samples from newly diagnosed metastatic patients, or from recurrent mCRC patients using the highly sensitive digital PCR technology, BEAMing (beads, emulsions, amplification, and magnetics), and compared with DNA sequencing data of respective FFPE (formalin‐fixed paraffin‐embedded) tumor samples. Discordant tissue RAS results were re‐examined by BEAMing, if possible. The prevalence of *RAS* mutations detected in plasma (51%) vs. tumor (53%) was similar, in accord with the known prevalence of RAS mutations observed in mCRC patient populations. The positive agreement between plasma and tumor RAS results was 90.4% (47/52), the negative agreement was 93.5% (43/46), and the overall agreement (concordance) was 91.8% (90/98). The high concordance of plasma and tissue results demonstrates that blood‐based *RAS* mutation testing is a viable alternative to tissue‐based RAS testing.

**Abbreviations**
BEAMing: beads, emulsions, amplification, and magnetics
CRC: colorectal cancer
ctDNA: circulating tumor DNA
EGFR: epidermal growth factor receptor
FFPE: formalin‐fixed paraffin‐embedded
LoB: limits of blank
LOD: limits of detection
MAF: mutant allelic fraction
mCRC: metastatic colorectal cancer
NPA: negative percent agreement
PPA: positive percent agreement
SOC: standard of care
WT: wild‐type

## 1 Introduction

Colorectal cancer (CRC) is a major health problem with ~1.3 million new cases diagnosed annually (‘International Agency for Research on Cancer. GLOBOCAN 2012: estimated cancer incidence, mortality, and prevalence worldwide in 2012’, n.d.). The disease stage at diagnosis is a significant prognostic factor, and in spite of global screening efforts, ~50% of patients will either present with, or relapse with metastatic disease (Kievit, 2002; Siegel *et al*., 2015). A key priority for patients with metastatic colorectal cancer (mCRC) is the implementation of an appropriate first‐line treatment strategy. Numerous randomized controlled trials have demonstrated the benefit of treating mCRC patients with monoclonal antibodies targeting the epidermal growth factor receptor (EGFR), such as cetuximab and panitumumab (Jonker *et al*., 2007; Saltz *et al*., 2004; Tabernero *et al*., 2007; Van Cutsem *et al*., 2007). The identification of patients wild‐type (WT) for *KRAS* codons 12 and 13 mutations increased response rates to anti‐EGFR therapy by as much as 60% (Douillard *et al*., 2010; Van Cutsem *et al.,* 2009a) and reinforced the approach of incorporating molecular diagnostics into clinical practice. Recent trials have demonstrated that a more comprehensive evaluation of *RAS*, so‐called *expanded RAS*, to include *KRAS* and *NRAS* codons 12, 13, 59, 61, 117, 146 can more precisely identify patients with mCRC for anti‐EGFR therapy than *KRAS* codons 12 and 13 testing alone (Bokemeyer *et al*., 2014; Douillard *et al*., 2013; Heinemann *et al*., 2014; Peeters *et al*., 2014).

Genotyping of tumor tissue can present challenges to even the most advanced clinical practice. Studies evaluating the genomic profiles of primary tumors and metastases have shown discordant results, attributed largely to molecular inter‐ and intratumor/metastasis heterogeneity (De Mattos‐Arruda *et al*., 2014; Gerlinger *et al*., 2012; Mao *et al*., 2015). Operationally, an optimal RAS testing procedure for biopsy and surgical resection specimens has yet to be uniformly established (Allegra *et al*., 2009; van Krieken *et al*., 2008; Tack *et al*., 2015). As an alternative and complement to tumor tissue genotyping, analysis of tumor DNA derived from plasma can provide a rapid genotype result, which accurately reflects the mutation status of tumor tissue (Bettegowda *et al*., 2014; Diehl *et al*., 2008). A unique feature of blood‐based genotyping is its potential to provide an integrative and gene mutation‐specific highly sensitive molecular analysis of an individual patient's tumor and/or metastases (Bettegowda *et al*., 2014; Diaz and Bardelli, 2014), thus eliminating sampling bias related to tissue heterogeneity.

Blood‐based tumor genotyping derives from observations that patients with cancer have markedly higher concentrations of circulating cell‐free DNA (cfDNA) than healthy individuals (Stroun *et al*., 1987). In patients with metastatic cancer, plasma‐derived ctDNA has been shown to be a reliable surrogate for genomic alterations in tumor tissue (Bettegowda *et al*., 2014; Diehl *et al*., 2008; Morelli *et al*., 2015). Among the various plasma ctDNA assays, BEAMing (beads, emulsions, amplification, and magnetics), based on emulsion digital PCR, has been shown to exhibit high sensitivity, enabling the detection of one mutant allele within a background of 10 000 wild‐type alleles (Diehl *et al*., 2006; Dressman *et al*., 2003; Li *et al*., 2006). In clinical trials, BEAMing has been extensively validated to assess tumor mutation status from the blood of patients with mCRC (Bettegowda *et al*., 2014; Morelli *et al*., 2015; Tabernero *et al*., 2015), with exemplary performance for the accurate assessment of expanded *RAS* (Bokemeyer *et al*., 2015; Van Cutsem *et al*., 2015; Venook *et al*., 2014). The objective of this study was to demonstrate the utility of a standardized blood‐based *RAS* genotyping system as an alternative to tissue‐based RAS genotyping prior to treatment with anti‐EGFR therapy.

## Materials and methods

### Study design

Two separate cohorts of advanced CRC patients from Australia and Germany were evaluated for concordance of *RAS* mutation status between plasma and tissue. A single blood sample from each patient was obtained immediately prior to biopsy or resection of tumors from either primary or metastatic sites. *RAS* mutation analysis of plasma was compared with the standard‐of‐care (SOC) tumor *RAS* testing performed on a primary or metastatic specimen (FFPE tumor tissue) from the same patient. In instances of discrepant *RAS* results between plasma and tissue, repeat *RAS* mutation testing was performed using BEAMing applied to the same FFPE tumor block as that used for SOC *RAS* testing. To determine concordance of plasma vs tissue *RAS* testing results, positive percent agreement (PPA), negative percent agreement (NPA), and overall percent agreement (OA) were calculated. In cases where SOC testing resulted in a WT determination and tissue BEAMing analysis revealed a *RAS* mutation, the BEAMing result was favored if the fraction of mutant alleles exceeded the SOC cutoff of 2%. Histopathology was performed and CEA levels were determined by the pathology and diagnostic laboratories at each hospital, respectively.

### Patients and samples

The local ethical committees approved sample collection, and consent was obtained for plasma analysis prior to tumor biopsy or resection (ethical votes Australia: Melbourne 03/90, Newcastle 11/04/20/4.03; ethical votes Germany: Munich 1926/07; Bochum 16‐5683). Collected patient characteristics included age, gender, disease status, treatment history, CEA concentration if available, histopathology and tumor staging. Overall, 98 patients were included in the concordance analysis. Four patient cases were excluded, with three patient plasma samples exhibiting inadequate plasma‐derived DNA for analysis and one patient for whom a *RAS* mutation result could not be confirmed in the original FFPE specimen when re‐evaluated by DNA sequencing.

The Australian cohort was comprised of 32 CRC patients having advanced disease (stage IV, or stage III with multiple lymph nodes affected). All FFPE tissue and plasma samples originated from patients at the John Hunter Hospital in Newcastle, New South Wales, or the Peter MacCallum Cancer Centre in East Melbourne, Victoria, Australia. The majority of patients (*n* = 24) presented with recurrent metastatic disease for whom tissue was obtained from the metastatic lesion. Eight patients with stage III disease and involvement of multiple (>2) lymph nodes were also evaluated.

The German cohort (*n* = 66) was comprised of 61 newly diagnosed and five mCRC patients with recurrent disease. All FFPE samples and accompanying *RAS* testing results were provided by the Medical University of Bochum Hospital, Bochum, and the University Hospital Klinikum rechts der Isar, Munich, Germany. In contrast to those in the Australian cohort, tumor samples from Germany were comprised largely of primary tumors obtained at first diagnosis of mCRC.

For patients in both cohorts, plasma samples were prepared from blood collected in K_2_‐EDTA Vacutainer^®^ tubes (Becton Dickinson, Franklin Lakes, NJ, USA) within 4 h of phlebotomy according to approved procedures for ctDNA analyses including a centrifugation step to pellet any cell debris (Sysmex Inostics GmbH, Hamburg Germany). All plasma samples were stored and shipped as 1 mL aliquots at −80 °C. Approximately 2 mL of plasma from each sample was thawed at room temperature for 10 min prior to ctDNA isolation. Purification of DNA from plasma was performed using the QIAamp DNA purification kit (Qiagen, Venlo, the Netherlands) according to the manufacturer's instructions. The total amount of human genomic DNA purified from plasma samples was quantified using a modified version of LINE‐1 real‐time PCR assay (Diehl *et al*., 2008). Any samples with total genome equivalents (GE) below 500 GE were deemed insufficient for mutational analysis, according to standard operating procedures (Sysmex Inostics GmbH).

### Plasma RAS mutation testing using the BEAMing method

Plasma samples were analyzed for 33 mutations in *KRAS* and *NRAS exons 2, 3, 4* by BEAMing at Sysmex Inostics. BEAMing utilizes emulsion digital PCR performed on magnetic beads to amplify single DNA molecules. Individual beads are then hybridized to allele‐specific fluorescently labeled probes complementary to the mutant and wild‐type DNA sequences. Finally, the bead population is analyzed by flow cytometry to count and sort wild‐type and mutant beads. The result is reported as the fractional abundance of mutant DNA alleles relative to wild‐type DNA alleles in a plasma sample. To generate the ratio of mutant to wild‐type DNA alleles (mutant allelic fraction, MAF), an average of 3 × 10^6^ beads are interrogated in each BEAMing analysis (approximately 90 000 beads per mutation). The absolute number of *RAS*‐mutant alleles is not reported by BEAMing as the determination of mutant status is dependent on the total amount of DNA in an individual sample. Total circulating DNA levels (both wild‐type and mutant) are subject to interpatient variability, which may be directly related to tumor burden or other characteristics such as inflammation and immune response.

In this study, the cutoff for the BEAMing *RAS* assay was 0.02%. Although it has been shown that BEAMing can detect one mutant molecule in a background of 10 000 wild‐type molecules (0.01%), the setting of the cutoff to 0.02% ensured that the limits of detection (LODs) for each of the 33 *RAS* mutations in the BEAMing *RAS* assay were well above background signals or limits of blank (LoBs) for each analyte to be detected in clinical samples. LODs were determined by probit regression analyses by spiking wild‐type (non‐*RAS* mutation‐containing) plasma samples with each *RAS* analyte. Background signals (LoBs) were determined in DNA prepared from wild‐type plasma samples lacking *RAS* mutations at low, medium, and high concentrations of genomic DNA. Based on the results of these experiments, the cutoff of 0.02% was observed to be appropriate so as to obtain a 95% probability/confidence interval of reporting a ‘mutation detected’ result (Sysmex Inostics GmbH, internal validation).

### Tissue *RAS* mutation testing

Formalin‐fixed paraffin‐embedded specimens were evaluated for *RAS* mutations in *KRAS* and *NRAS* exons 2, 3, and 4 using pyrosequencing, Sanger sequencing, or next‐generation sequencing according to the procedures established in routine clinical use at participating institutions. The cutoff threshold *RAS* MAF for calling a specimen mutant as validated at the local institutions was 2% and 5% for Australian and German cohorts, respectively, with pyrosequencing utilized for all German specimens. For cases where only *KRAS* exon 2 was evaluated by the SOC method, tissue specimens were re‐examined for expanded *RAS* either by DNA sequencing at the provider's institution or by BEAMing. BEAMing of tissue samples was also used to re‐evaluate the result provided by the standard‐of‐care assay for cases where the BEAMing plasma and SOC tissue results were discordant. The cutoff for tissue BEAMing was set at 1.0% MAF as demonstrated in CRYSTAL and OPUS studies (Bokemeyer *et al*., 2015; Van Cutsem *et al*., 2015).

### Statistical analyses

Concordance of *RAS* mutation status was determined by calculating the agreement of *RAS*‐mutant and WT cases. Fisher's exact test was used to assess the significance of relationships for plasma and tissue *RAS* results. All statistical tests were two‐sided; the threshold for statistical significance was *P* < 0.05. MAF values for newly diagnosed vs recurrent mCRC patients were evaluated by calculating mean MAF values with standard errors and compared with p values derived using Welch's unequal variances t‐test. Correlation between ctDNA and CEA levels was assessed using Pearson's rank correlation.

## Results

### Patient characteristics

In total, 98 patients with histologically confirmed metastatic or stage III CRC with multiple lymph node involvement (54 males and 44 females having a median age of 66 years) were evaluated. Staging was based on postoperative histopathology and imaging diagnoses. The baseline characteristics of patients are shown in Table 1. At the time of study inclusion, the majority of patients (91.8%) had stage IV colorectal adenocarcinoma. Most patients (70/98) had newly diagnosed disease and were naïve to treatment (71.4%). With respect to treatment, ~ one‐third of the patients had recurrent disease and received at least one line of prior chemotherapy. The colorectum was the predominant site of tissue biopsy (78%). In patients for whom a metastatic site was submitted for *RAS* mutation analysis (22%), the most frequent site was the liver (77%) followed by the lung (18%).

**Table 1:** Patient characteristics

Patients	98
Median age (range)	66 (21–92)
Gender	
Female, *n* (%)	44 (44.9%)
Male, *n* (%)	54 (55.1%)
Disease status at time of biopsy, *n* (%)	
Stage III, newly diagnosed	8 (8.2%)
Stage IV, newly diagnosed	62 (63.3%)
Stage IV, recurrent disease	28 (28.6%)
Site of tissue biopsy, *n* (%)	
Primary tumor	76 (78%)
Metastases	22 (22%)
Liver	18 (82%)
Lung	4 (18%)
Therapeutic history, *n* (%)	
Treatment naive	70 (71.4%)
≥first line of therapy	28 (28.6%)
Baseline serum CEA, median (IQR) (*n* = 65)	18.4 (6.25–60.25)

Levels of CEA in blood samples were available for 65 of 98 patients in the concordance analysis. The median CEA concentration from these 65 patients was 18.40 ng·mL^−1^ (range: 2–4069; mean ± SD 193.6 ± 624.6). A comparison of CEA concentrations in newly diagnosed stage IV patients versus those diagnosed with metastatic disease at recurrence was then made. The median CEA concentrations in newly diagnosed stage IV patients and those with recurrent disease were 19.40 ng·mL^−1^ (mean ± SD 236.3 ± 700.0) and 9.90 ng·mL^−1^ (mean ± SD 37.86 ± 52.26), respectively. A two‐tailed *t*‐test of CEA levels indicated that differences in CEA levels between these two patient groups were at the threshold of statistical significance (*P* = 0.0504).

### *RAS* mutation status analysis from plasma and tissue

*RAS* mutation analysis in plasma was performed using the BEAMing expanded *RAS* mutation panel (Table 2), which detects 33 mutations encoding pathogenic variants of KRAS and NRAS proteins. *RAS* mutation status was evaluable in both plasma and tissue of all 94 patients. Overall, *RAS* mutations were detected in 53% of tumor tissue samples and in 51% of plasma samples (Table 3). The frequency of *RAS* mutations in patients investigated in this study was in agreement with the results of other groups performing expanded *RAS* analysis (Sorich *et al*., 2015). The vast majority of mutations detected by both plasma and tissue methods were *KRAS* codons 12 and 13 (Table 3). *RAS* codon 61 mutations were detected in only four cases.

**Table 2:** Individual *KRAS* and *NRAS* mutations detected by BEAMing

KRAS		NRAS	
Exon	Mutation	Exon	Mutation
2	G12S G12R G12C G12D G12A G12V G13D	2	G12S G12R G12C G12D G12A G12V G13D G13R G13V
3	A59T Q61L Q61H^a^ Q61H^a^	3	A59T Q61L Q61H^a^ Q61H^a^ Q61K Q61R
4	K117N^a^ K117N^a^ A146T A146V	4	K117N^a^ K117N^a^ A146T

a Denotes two separate mutations detected for each of these codons.

**Table 3:** RAS mutation by exon/codon: frequency and prevalence

RAS mutation	Tissue		Plasma	
	*N*	%	*N*	%
KRAS Exon 2 Codon 12	40	77	35	70
KRAS Exon 2 Codon 13	10	19	10	20
KRAS Exon 3 Codon 61	1	2	1	2
NRAS Exon 3 Codon 61	1	2	3	6
KRAS Exon 4 Codon 146	1	2	1	2
RAS prevalence	52/98 = 53.1%		50/98 = 51.0%	
WT prevalence	46/98 = 46.9%		48/98 = 49.0%	

The concordance of *RAS* status between matched plasma and tissue from each patient is summarized in Table 4. The *RAS* mutation status determined by BEAMing from plasma versus the reference method was concordant in 90 of 98 cases examined (91.8% OPA). *RAS* mutations were found in 47 of 52 cases tested (90.4% PPA). Of 46 patients identified as WT by tissue testing, 43 were also found to be WT in plasma (93.5% NPA). When considering only patients with mCRC, plasma *RAS* mutations were found in 45 of 49 cases (91.8% PPA), no *RAS* mutations were found in 38 of 41 cases (92.7% NPA), and *RAS* mutation status was concordant in 83 of 90 cases (92.2% OPA). Initially, nine discrepant *RAS* mutation results were found among the 98 cases. Five samples evaluated by tissue testing were *RAS mutation+,* whereas the corresponding plasma samples were WT. Conversely, plasma analysis revealed *RAS* mutations in four patients whose tumors were determined WT by tissue testing.

**Table 4:** Concordance of plasma and tissue RAS mutation results

	Tumor tissue RAS result						
	RAS	Mutant	WT	Total	PPA (95% CI)	NPA (95% CI)	OPA (95% CI)
Plasma ctDNA RAS result	Mutant	47	3	50	100 × 47/52 = 90.4% (79%, 96%)	100 × 43/46 = 93.5% (82%, 98%)	100 × 90/98 = 91.8% (85%, 96%)
	WT	5	43	48			
	Total	52	46	98			

### Discordance analysis

To evaluate discrepancies between the results of matched tumor and plasma samples, tissue BEAMing was employed as an orthogonal assay. Previous studies have shown that BEAMing is an accurate technique for the mutational analysis of archival FFPE tumor tissue (Bokemeyer *et al*., 2015; Morelli *et al*., 2015; Van Cutsem *et al*., 2015). Whenever possible, FFPE samples matched to the date of surgery and tissue site for cases having discrepant results were re‐analyzed by BEAMing. All but three FFPE specimens were either unavailable or exhibited severely degraded DNA and could not be tested. Of the three samples adequate for re‐analysis, tissue BEAMing confirmed the results of SOC tissue testing in two cases and the results of plasma testing in one case (Table 5). In the two cases in which SOC tissue results were confirmed, BEAMing detected a result of WT in one and a *KRAS* codon 12 mutation in the other. However, in one case, re‐examination with tissue BEAMing confirmed a *KRAS* G12D mutation (2.86% MAF) also detected in the plasma, but not detected by SOC tissue testing.

**Table 5:** Discordant analysis. In cases where no tissue re‐evaluation was possible, the final call remained discordant

Sample ID	Stage	Site of tissue biopsy	Tissue result	Plasma result	Plasma MAF%	Tissue re‐evaluation	Final call	CEA (ng·mL^−1^)
AUS007	IV	MET (lung)	**KRAS** **G12C**	WT		**KRAS** **G12C (12.1%)**	P‐FN	2.4
AUS030	IIIB	Primary	KRAS G12D	WT		Low DNA/NA	Discordant	Not available
GER010	IV	Primary	WT	NRAS Q61R	0.258%	NTA	Discordant	1452
GER016	IV	Primary	KRAS G12D	WT		NTA	Discordant	18.4
GER024	IV	Primary	WT	NRAS Q61R	0.237%	NTA	Discordant	7.1
GER028	IV	Primary	WT	**KRAS** **G12D**	0.425%	**KRAS** **G12D (2.9%)**	**Concordant**	27.5
GER029	IV	Primary	KRAS G12D	WT		NTA	Discordant	41.4
GER051	IV	Primary	KRAS G12V	WT		NTA	Discordant	42.6
GER056	IV	Primary	**WT**	KRAS G12A	0.111%	**WT**	P‐FP	4.52

WT, wild‐type; NA, nonanalyzable; NTA, no tissue available; P‐FN, plasma false‐negative; P‐FP, plasma false‐positive.

Comparisons of RAS mutation results obtained by BEAMing analyses of tissue and those obtained by the SOC tissue test are designated in boldface type.

As a patient's CEA concentration is used as a prognostic indicator of disease status, we examined whether CEA concentration may be related to the likelihood of detecting a plasma mutation that might explain discordance in the eight stage IV patients (e.g., low CEA levels correspond to undetectable ctDNA). As shown in Table 5, one patient with a *KRAS* codon 12 mutation in the tumor but showing no detectable *RAS* mutation in plasma had a normal CEA concentration (2.4 ng·mL^−1^). The median CEA concentration in patients for whom a mutation was detected in plasma, but not in the tissue, was 17.3 ng·mL^−1^. In patients for whom a mutation was present in the tissue, but not detected in plasma, the median CEA concentration was 29.9 ng·mL^−1^. Based on the available samples, we did not observe any correlation between CEA levels and the ability to detect *RAS* mutations.

### Plasma mutant allele frequency and correlation with tumor burden

**Figure 1:**
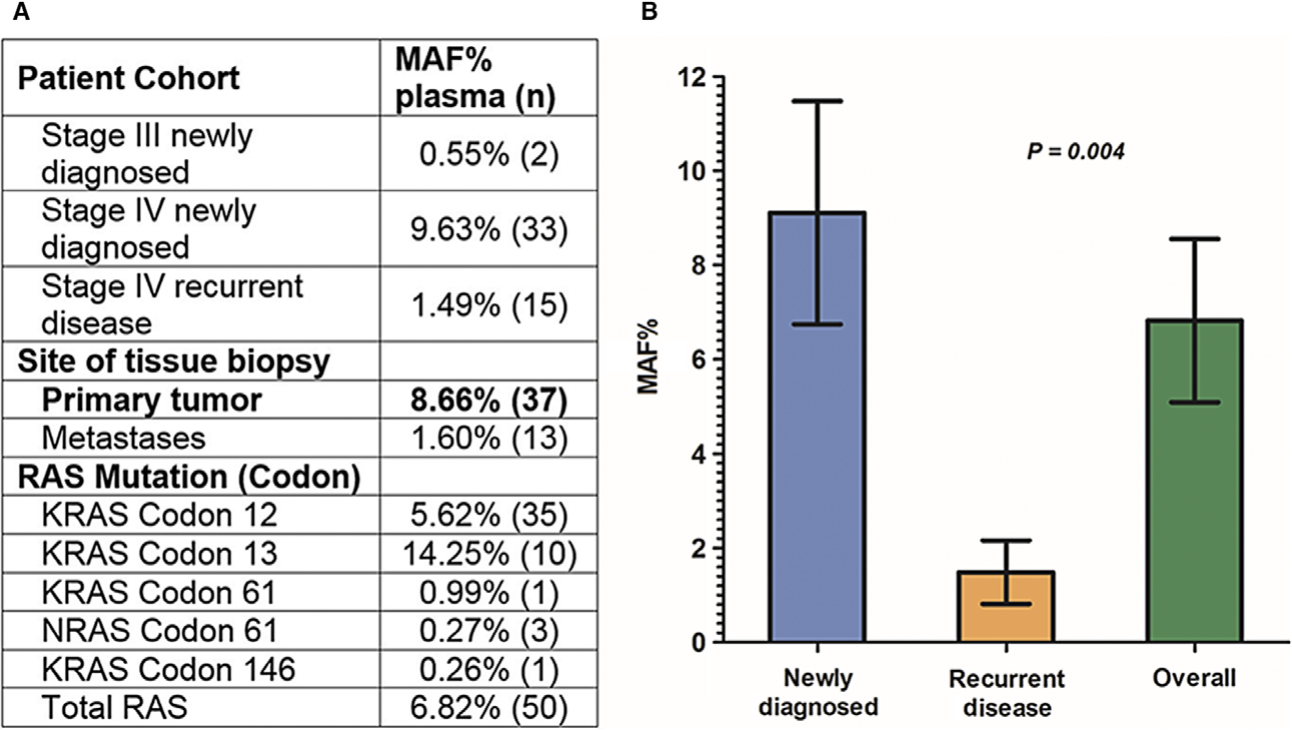
(A) Bar chart showing the average plasma DNA‐mutant fractions detected in ctDNA of patients with newly diagnosed compared to recurrent disease as well as the overall cohort of patients with RAS mutations detected in the plasma by BEAMing. (B) Average value and standard error for all patients (stage III and stage IV), those with newly diagnosed and recurrent disease are 6.82 ± 1.73, 9.11 ± 2.36, and 1.49 ± 0.67, respectively. *P* = 0.004 for MAF% in newly diagnosed patients compared to those with recurrent disease. *P* values were derived from a Welch's unequal variances *t*‐test.

A unique feature of BEAMing ctDNA analysis is the ability to determine the fraction of cell‐free mutant alleles as a proportion of the overall cell‐free DNA content in circulation at the time of sampling. For the 50 patients with detectable plasma *RAS* mutations, the average MAF was 6.82% (Fig. 1). Given the possibility that the frequency of circulating mutant alleles is related to overall tumor burden or extent of metastatic invasion, MAF values in plasma were compared between newly diagnosed mCRC patients and those having metastatic recurrence. A statistically significant relationship was observed between the patient clinical diagnosis status and mean proportion of mutant *RAS* alleles in circulation. In stage IV newly diagnosed patients with intact primary tumors of the colorectum, the MAF was 6.5‐fold higher (9.63%) compared with those patients who presented with recurrent disease after removal of their primary tumors (1.49%) (*P* = 0.0055, Fig. 1). In patients presenting with stage III newly diagnosed disease, the average MAF was also lower (0.55%); this is in line with results of previous reports that earlier‐stage tumors tend to release lower amounts of tumor DNA into circulation (Bettegowda *et al*., 2014). There appeared to be no correlation between CEA concentration and MAF among those patients whose CEA levels were available at the time of blood collection for ctDNA analysis (Pearson's *r* = 0.231, *P* = 0.1822).

## Discussion

The accurate prescription of anti‐EGFR therapy is of high clinical importance for patients with mCRC. Retrospective analyses of data from randomized controlled clinical trials consistently demonstrate that *KRAS* exon 2 mutation is a contraindication for the administration of anti‐EGFR therapy, regardless of the chemotherapy backbone (Amado *et al*., 2008; Bokemeyer *et al*., 2011; Douillard *et al*., 2010; Karapetis *et al*., 2008; Peeters *et al*., 2010; Van Cutsem *et al*., 2011). In 2009, *KRAS* testing was thus established in both European and US clinical practice guidelines as a predictive marker of response to anti‐EGFR therapy (Allegra *et al*., 2009; Benson *et al*., 2014; Van Cutsem *et al.,* 2009a, b). With *KRAS* exon 2 mutation status widely accepted as a predictor for a lack of response to anti‐EGFR therapy, it became clear that not all *KRAS* exon 2 wild‐type patients responded to treatment. Further refinement in biomarker testing was pursued to improve patient outcomes and avoid unnecessary treatment‐related side effects and costs, with a focus on *KRAS* exon 3 and 4 mutations—shown to confer resistance to EGFR antibodies similar to *KRAS* exon 2 mutations (Janakiraman *et al*., 2010; Smith *et al*., 2010). Subsequent evaluation of *NRAS* mutations revealed their occurrence in CRC tumors with persistent GTPase activity similar to alterations in *KRAS* (Irahara *et al*., 2010). Incorporation of expanded *RAS* testing into practice is expected to increase the proportion of patients ineligible for anti‐EGFR therapy from ~45% to ~55% (Sorich *et al*., 2015). Current clinical practice guidelines now recommend expanded *RAS* analysis be performed to more precisely identify patients for anti‐EGFR therapy (Allegra *et al*., 2016; Benson *et al*., 2014; Van Cutsem *et al*., 2014).

Phase III clinical trials utilizing the BEAMing expanded *RAS* mutation panel have shown superior overall survival for *RAS* WT vs. *RAS*‐mutant mCRC patients when treated in first line with EGFR antibodies (Bokemeyer *et al*., 2015; Van Cutsem *et al*., 2015; Venook *et al*., 2014). Notably, specimens from 548 patients with mCRC previously defined as *KRAS* exon 2 WT in CRYSTAL and OPUS studies re‐examined with BEAMing detected other *RAS* mutations in 14.7% and 26% of patients, respectively. These results validated the use of BEAMing to evaluate expanded *RAS* testing in order to select patients for anti‐EGFR therapy. Although great strides have been made, the standardization of *RAS* testing has been difficult to achieve, largely due to variability of testing methodologies and DNA quality and quantity of FFPE specimens. Implementation of reliable tumor tissue genotyping programs has also been challenging. For instance, a recent external quality assessment (EQA) revealed significant interlaboratory variability within routine approaches to testing of *RAS* in Europe (Tack *et al*., 2015). Moreover, obtaining suitable tumor tissue samples can pose a challenge—particularly in recurrent mCRC patients with distant metastases (Wang *et al*., 2010). At initial diagnosis, the timing of molecular testing results is of critical importance for first‐line treatment. However, in a recent study of patients with mCRC evaluated for first‐line therapy, ~25% of patients did not have *RAS* testing requested at or at least one month after their initial diagnosis of metastatic disease (Longin, 2015). These findings are supported by a survey of European physicians, which revealed that *RAS* testing turnaround times and the unavailability of tissue were the most frequent factors cited for treating mCRC patients with unknown *KRAS* status (Trojan *et al*., 2015). Moreover, a recent EQA survey found that half of all participating laboratories exceeded the required turnaround time of 14 days for *RAS* testing (Tack *et al*., 2015). This presents a challenge to the broad realization of individualizing therapy and providing an accurate blood‐based *RAS* mutation assay with rapid turnaroundtime would help circumvent these issues.

The primary objective of this study was to validate blood‐based *RAS* mutation analysis as an alternative to tissue‐based *RAS* mutation analysis prior to anti‐EGFR therapy. Two geographically isolated cohorts of patients with CRC from Australia and Germany were examined for concordance of *RAS* mutations in plasma and tissue. The Australian cohort mainly comprised patients with metastatic recurrences and was representative of the clinical practice of assessing eligibility for anti‐EGFR therapy following first‐line chemotherapy. In contrast, the German cohort was comprised predominantly of newly diagnosed, treatment‐naïve mCRC patients, a cohort eligible for first‐line anti‐EGFR therapy. The entire patient group in this study represented a reasonable cross‐section of the anti‐EGFR intended use population.

Previous concordance studies of SOC FFPE *KRAS* mutation detection assays showed minimal variation in the presence or absence of *KRAS* mutations, most likely due to differential tumor cell selection from formalin‐fixed tumor tissue (Whitehall *et al*., 2009). Overall, the concordance between plasma and tissue *RAS* mutation status determined in this study was 93% and suggests that plasma *RAS* mutation detection is as good as tissue‐based detection strategies, but has the advantage that it does not rely on the testing of DNA isolated from fixed tissue. The most notable difference was the threshold cutoffs for *RAS* positivity between the two sites. Due to this variability, any tissue samples exhibiting *RAS* mutation status that differed from plasma were, if available, re‐examined by BEAMing. For one such case, BEAMing identified the same *KRAS* mutation in tissue that was identified in plasma, contrary to the original SOC *RAS* WT result. Interestingly, the MAF obtained by tissue BEAMing was 2.86%, falling between the 2% and 5% cutoffs of the SOC methods. This circumstance highlights the variability in SOC RAS testing techniques, and with the possibility to apply an orthogonal assay, this case was determined to be concordant. These comparisons also suggest that further improvements in the agreement of plasma and tissue *RAS* testing results may be achieved when both the methods of plasma preparation and FFPE *RAS* testing are standardized.

A systemic assessment of mutation status in a patient with metastatic CRC represents a key advantage of blood‐based testing. However, in a patient with widely metastatic disease, blood‐based mutation testing does not yet have the ability to discern the site of ctDNA origin. In our study for example, three patients had a *RAS* mutation that was detected in plasma, but not in the primary colorectal tumor. All three patients were newly diagnosed, treatment naïve with intact primary tumors, and presented with hepatic metastases; one patient had additional pulmonary metastases. As all three patients presented with distant metastases at the time of blood draw, a reasonable explanation of discordance may be attributed to heterogeneity of the genotype between metastatic and primary tumors. Indeed, several studies evaluating intertumor molecular heterogeneity between primary tumors and metastases in the same patient have shown mutational discordance in 3.6–32.4% of cases (Artale *et al*., 2008; Italiano *et al*., 2010; Kim *et al*., 2012; Knijn *et al*., 2011; Tie *et al*., 2011). As we have observed a lower plasma *RAS* MAF in patients with recurrent metastatic disease (Fig. 1), a tenable hypothesis is that in these three newly diagnosed patients lacking *RAS* mutations in their primary tumor, but having low plasma *RAS* MAF (0.111–0.258%), the *RAS* ctDNA in these three patients may be contributed by a single or few metastatic sites.

## Conclusions

Determination of a patient's *RAS* mutational status from plasma may provide distinct advantages compared to *RAS* testing of FFPE samples. A specific application with future clinical utility may be the routine surveillance of plasma mutation status to assess RAS‐mediated resistance in patients receiving anti‐EGFR therapy. This approach has been shown to provide a more precise gauging of the efficacy/failure of anti‐EGFR therapy (Diaz *et al*., 2012; Morelli *et al*., 2015). Indeed, BEAMing has already revealed substantial differences in *RAS* mutation status between baseline mCRC tumor samples as compared with current plasma mutation status. This advantage was demonstrated in a clinical trial of 503 patients with mCRC that investigated whether plasma *RAS* mutation status was associated with response to regorafenib (Tabernero *et al*., 2015). In a cohort of patients whose archival tumor was *KRAS* WT and consequently received anti‐EGFR therapy, *KRAS* mutations were detected by BEAMing in the plasma of 48% of patients at disease progression. This exemplifies the use of plasma ctDNA testing to provide a real‐time assessment of mutation status. Blood‐based mutation assessment may therefore help define critical decision points for the individualized management of patients with CRC, providing a finer resolution of molecular cues to signal optimal timing for treatment hiatus and re‐initiation of targeted therapy.

A necessary first step toward implementing blood‐based *RAS* testing in clinical practice is to demonstrate concordance between ctDNA and tissue *RAS* mutation testing. The results presented herein provide a high level of confidence that the clinical performance of plasma *RAS* testing using BEAMing is comparable to FFPE tissue testing and can be useful in a clinical setting to select patients with mCRC for anti‐EGFR therapy.

## Author contributions

WS, RJS, PR, DLE, FSJ, SH, and SBF conceived and designed the project. SD, WL, CJM, PP, BD, SS, CH, HP, AL, ES, KN, TC, DA, SPC, AT, ARS, WU, CT, HW, JS, RV, HF, KPJ, UN, JSH, MP, DV, AB, BDD, SHB, FSJ, and SH acquired the data. WS, RJS, AT, DLE, FSJ, SH, and SBF analyzed and interpreted the data. WS, RJS, DLE, FSJ, SH, and SBF wrote the manuscript.

## Conflict of interest

P.R. is employee of the Merck KGAa; D.L.E. and F.S.J. are employees of the Sysmex Inostics Inc. W.S. has a nonprofit cooperation with Sysmex Inostics to use the BEAMing technology.
